# A Case of Swallowing Apraxia Due to Acute Infarct in the Right Precentral Gyrus

**DOI:** 10.7759/cureus.36119

**Published:** 2023-03-14

**Authors:** Ghulam Kawnayn, Humayun Kabir, Mahin Binte Anwar, Raihan Mahmud, Muhammad Rezeul Huq

**Affiliations:** 1 Neurology, Combined Military Hospital, Dhaka, BGD; 2 Radiology, Combined Military Hospital, Dhaka, BGD

**Keywords:** cerebral dominance, case report, dysphagia, stroke, swallowing hesitation, swallowing apraxia

## Abstract

Swallowing apraxia is an intriguing type of apraxia where the patient cannot swallow despite normal neurological examinations including motor, sensory and cerebellar function. In this case report, we present a 60-year-old hypertensive male with swallowing apraxia. There was no attempt to swallow when food materials were given inside his mouth. Although he had normal examination findings including intact lip, tongue, palatal movement, and gag reflex. His cognition was also intact, as he could follow simple commands accurately. Except for a small infarct in the right precentral gyrus in the MRI (Magnetic Resonance Imaging) of the brain, his investigation findings were normal. We treated him with nasogastric feeding, and he gradually recovered over a month. Clinicians should consider swallowing apraxia as one of the clinical features of stroke when patients present with acute onset of swallowing problems. This case report is believed to increase awareness regarding this condition and add valuable information to relevant further studies.

## Introduction

Apraxia can be defined as the inability to do learned motor activity despite intact motor, sensory, coordination, and comprehension [1]. Swallowing apraxia is one of the types characterized by a “lack of labial, lingual, and mandibular coordination during the oral stage with residual pooling of material on the tongue and palate” [2]. Though structural lesions of the cerebral hemisphere may lead to the development of this condition, stroke is the commonest cause [3-5]. Here we present a case of swallowing apraxia due to acute ischemic stroke with a lesion in the right precentral gyrus. There is a scarcity of case reports or other research works regarding this condition. As there is ongoing debate regarding definite clinical patterns and radiological findings in swallowing apraxia, this case report will add new information to the literature.

## Case presentation

A 60-year-old hypertensive male presented to us with sudden onset of difficulty in swallowing for one day. Except for high blood pressure (170/100 mm of Hg), other general physical examination findings were normal. He was conscious and oriented. GCS (Glasgow Coma Scale) score was full. All cranial nerves were intact including normal lip, tongue, palatal movement, taste sensation, and gag reflex. The speech was normal. We also found intact motor, sensory, and coordination systems in limb examination. His gait was intact. 

We assessed his swallowing function by offering both liquid and solid foods by teaspoon. The patient did not initiate the swallowing or chewing process but only kept the food inside the oral cavity. He also had excessive drooling of saliva. 

**Figure 1:**
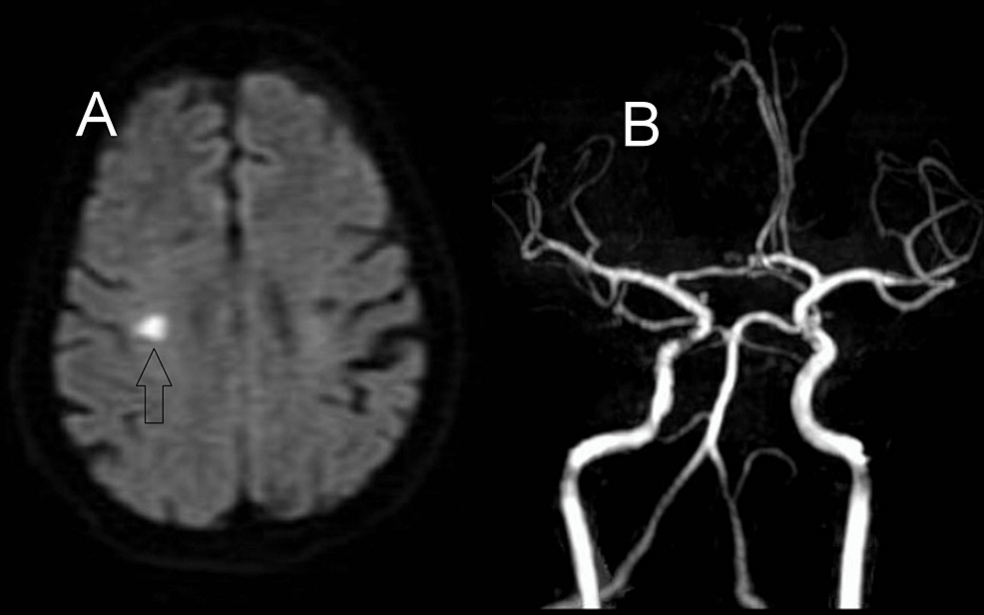
MRI and MRA study images (A) Magnetic resonance imaging (MRI) of the brain diffusion-weighted image (DWI) sequence showing acute infarct (arrow) in the right precentral gyrus. (B) The magnetic resonance angiography (MRA) of the brain was normal.

MRI (Magnetic Resonance Imaging) of the brain revealed a small infarct in the right precentral gyrus (Figure 1A). MRA (Magnetic Resonance Angiography) of the brain (Figure 1B) and neck vessels were normal. Electrocardiography and echocardiography revealed no abnormality. Other relevant investigations like diabetic profile, lipid profile, and thyroid function tests were within normal limits. We did 24 hours of Holter monitoring to exclude paroxysmal atrial fibrillation, which was also normal. Except for hypertension, we did not find any other reversible risk factors. We could not perform a videofluoroscopic swallowing assessment as our fluoroscope was out of order at that moment. The patient wanted to be discharged before the fluoroscope was functioning again, so he was discharged on request. However, from clinical assessment, we considered swallowing apraxia the most likely diagnosis.

We initially tried food of different textures and tastes, including sour foods, in a calm and quiet environment. The patient’s favorite dishes were given. A one-to-one caregiver was ensured. As these measures failed, we started nasogastric feeding and other supportive management including an antiplatelet, statin, and anti-hypertensive agents. The patient recovered gradually. On his follow-up visit after one month, we could successfully remove the nasogastric tube.

## Discussion

Swallowing hesitancy is another terminology that is sometimes synonymous with swallowing apraxia [6]. Recent literature suggests that swallowing apraxia or hesitation can be divided into two subtypes [3,6]. One is the rippling type associated with the involuntary rippling movement of the tongue due to repeated trial and error in deglutition [6]. In the second one, the stasis type, there is temporary stasis of the tongue [6]. We could not conclude about the definite subtype in our case due to the lack of a videofluoroscopic study. A patient with swallowing apraxia may have additional features like buccofacial apraxia or apathy [3,6]. We did not find any additional abnormalities in our case.

Most of the research works suggest left-sided hemispheric lesions causing swallowing apraxia [3,6]. Cerebral dominance was found to be associated with a rippling type of swallowing apraxia where left-sided cerebral pathology was usually found [6]. Lesions are found in the precentral gyrus or surrounding area. In the stasis type, lesions are found in broader areas of the left cerebral hemisphere including the primary motor cortex, orbitofrontal cortex, middle frontal gyrus, inferior frontal gyrus, and insula [6]. In our case, the patient had a right-sided lesion in the precentral gyrus. More research works are needed to confirm whether swallowing apraxia is associated with cerebral dominance or not. 

The management of swallowing apraxia is yet not established. Compensatory swallowing techniques like neck extension may help in the oral phase of swallowing but are not recommended due to the risk of asphyxia [4]. Transcranial direct current stimulation is another recommended treatment option [7]. Sour foods are preferred while giving oral feeding, as sour foods facilitate the involuntary part of the swallowing reflex [5]. Maintenance of nutrition is essential and nasogastric or percutaneous gastro-jejunostomy tubes are often needed [4]. The prognosis is usually favorable [6]. In our case, we treated the patient with nasogastric tube feeding and he recovered gradually.

Our case has some limitations, as we could not assess swallowing function formally by videofluoroscopy. However, his clinical profile, including the bedside swallow test, was suggestive of swallowing apraxia.

## Conclusions

In the present case, the patient could not initiate swallowing when food was given inside the oral cavity. He had normal lip, tongue, and palatal movement when the oral cavity was empty. Swallowing apraxia was diagnosed on clinical grounds. Clinicians should be vigilant about swallowing apraxia as a possible cause of swallowing difficulties in a stroke patient. More research is needed regarding the proper evaluation and management of this condition.
